# Performance and Cost Efficiency of *KRAS* Mutation Testing for Metastatic Colorectal Cancer in Routine Diagnosis: The MOKAECM Study, a Nationwide Experience

**DOI:** 10.1371/journal.pone.0068945

**Published:** 2013-07-25

**Authors:** Hélène Blons, Etienne Rouleau, Nathanaël Charrier, Gilles Chatellier, Jean-François Côté, Jean-Christophe Pages, Florence de Fraipont, Jean-Christophe Boyer, Jean Philippe Merlio, Alain Morel, Marie-Claude Gorisse, Patricia de Cremoux, Karen Leroy, Gérard Milano, L’Houcine Ouafik, Jean-Louis Merlin, Delphine Le Corre, Pascaline Aucouturier, Jean-Christophe Sabourin, Frédérique Nowak, Thierry Frebourg, Jean-François Emile, Isabelle Durand-Zaleski, Pierre Laurent-Puig, on behalf of the MOKAECM collaborative group

**Affiliations:** 1 UMR-S775, INSERM, Paris, France; 2 Université Paris Descartes, Paris, France; 3 Assistance Publique Hôpitaux de Paris Department of biology, Hôpital Européen Georges Pompidou, Paris, France; 4 Institut Curie, Paris, France; 5 Assistance Publique Hôpitaux de Paris, Henri Mondor-Albert Chenevier Hospitals, Department of Public Health, Creteil; URCEco Ile de France, Paris, France; 6 Assistance Publique Hôpitaux de Paris, Hôpital Européen Georges Pompidou, URC Paris, France; 7 EA4340, Université de Versailles St Quentin en Yvelines; Assistance Publique Hôpitaux de Paris Hôpital Ambroise Paré, Department of Pathology; Boulogne-Billancourt, France; 8 INSERM U966, Université François Rabelais de Tours, Faculté de Médecine, Tours, France; 9 Grenoble University Hospital Department of biology, Grenoble, France; 10 Nîmes University Hospital, Department of Biochemistry, Nîmes, France; 11 Bordeaux University Hospital Segalene Department of Tumor Biology, Pessac; Bordeaux University EA 2406, Bordeaux, France; 12 Cancer Center Paul Papin; INSERM U892; University of Angers, Angers, France; 13 Institut Jean Godinot, Reims, France; 14 Assistance Publique Hôpitaux de Paris, Saint Louis Hospital, Molecular oncology unit, Department of biochemistry, Paris, France; 15 Assistance Publique Hôpitaux de Paris Department of pathology Henri Mondor Hospital, Creteil, France; 16 Institut Laccassagne, Nice, France; 17 Aix-Marseille Université, Inserm-CRO2 UMR911, AP-HM, CHU Nord, Service de Transfert d’Oncologie Biologique, Marseille, France; 18 Centre Alexis Vautrin, Vandoeuvre-les-Nancy, France; 19 Rouen University Hospital Department of surgical and molecular pathology; Inserm U1079, Rouen, France; 20 INCa Institut National du Cancer, Boulogne-Billancourt, France; 21 Rouen University Hospital Department of Genetics, Inserm U614, Rouen, France; The Chinese University of Hong Kong, Hong Kong

## Abstract

**Purpose:**

Rapid advances in the understanding of cancer biology have transformed drug development thus leading to the approval of targeted therapies and to the development of molecular tests to select patients that will respond to treatments. *KRAS* status has emerged as a negative predictor of clinical benefit from anti-EGFR antibodies in colorectal cancer, and anti-EGFR antibodies use was limited to *KRAS* wild type tumors. In order to ensure wide access to tumor molecular profiling, the French National Cancer Institute (INCa) has set up a national network of 28 regional molecular genetics centers. Concurrently, a nationwide external quality assessment for *KRAS* testing (MOKAECM) was granted to analyze reproducibility and costs.

**Methods:**

96 cell-line DNAs and 24 DNA samples from paraffin embedded tumor tissues were sent to 40 French laboratories. A total of 5448 *KRAS* results were collected and analyzed and a micro-costing study was performed on sites for 5 common methods by an independent team of health economists.

**Results:**

This work provided a baseline picture of the accuracy and reliability of *KRAS* analysis in routine testing conditions at a nationwide level. Inter-laboratory Kappa values were >0.8 for *KRAS* results despite differences detection methods and the use of in-house technologies. Specificity was excellent with only one false positive in 1128 FFPE data, and sensitivity was higher for targeted techniques as compared to Sanger sequencing based methods that were dependent upon local expertise. Estimated reagent costs per patient ranged from €5.5 to €19.0.

**Conclusion:**

The INCa has set-up a network of public laboratories dedicated to molecular oncology tests. Our results showed almost perfect agreements in *KRAS* testing at a nationwide level despite different testing methods ensuring a cost-effective equal access to personalized colorectal cancer treatment.

## Introduction

New therapeutic approaches such as anti-EGFR targeted therapies and concurrent identification of molecular biomarkers to identify sub-groups of potentially responsive tumors had created a need for routine molecular characterization of cancers. In colorectal cancer, the demonstration that patients with *KRAS* mutated tumors did not benefit from anti-EGFR monoclonal antibodies was established independently of the technology used to identify *KRAS* mutated tumors [1]. This result was rapidly followed by a directive of the European Medicines Agency (EMEA) that restricted the use of cetuximab (Erbitux®) and panitumumab (Vectibix®) to patients with *KRAS* wild-type metastatic colorectal cancer [2].

With more than 940,000 new colorectal cancer cases worldwide each year, the use of anti-EGFR targeted therapies are faced with main issues, an economical one: who pays for the test or the drugs and a medical one: who performs the test? The French public health insurance system decided to provide targeted therapy for colorectal cancer in line with the EMEA recommendation. In parallel, the French government and the National Cancer Institute (INCa) have set up a national network of 28 regional molecular genetics centers to implement routine molecular testing for colorectal cancer. More than one laboratory can be related to one regional center.

Each laboratory developed *KRAS* testing according to its own expertise and to the locally available instruments. The number of tests increased from 1,100 in 2007 to 10,012 in 2008 and 17,246 in 2009. From then on, the number of tests was stable and covered the expected incidence of metastatic colorectal cancer patients in France. A founding of €2.5M was devoted to *KRAS* testing. This organization seemed cost-effective considering global gain on drug costs. It was necessary to prove that *KRAS* testing results were reproducible between molecular laboratories. Each laboratory using one or more genotyping method was evaluated by an external quality control program, the multicenter program: *KRAS* Oncogene Mutation detection in the treatment of Metastastic Colorectal cancer by EGFR Antibodies (MOKAECM). The MOKAECM project was set up as an external quality control and laboratories were free to choose and develop their own method for *KRAS* testing.

Previous comparative studies evaluated one technology [3] ^,^ [4], [5]. Others compared different techniques with one tested technology per site. In both cases the robustness of a technology used with different levels of expertise cannot be evaluated [6] [7]. A national assessment of *KRAS* mutation testing linking actual practices associated with cost evaluation has never been done up to now.

The first objective of the MOKAECM project was to evaluate at a nationwide level the performance of *KRAS* testing for clinical purpose (sensitivity and reproducibility). The second was to estimate and compare the costs associated to each technology. As this study covers a national territory including all the INCa labeled molecular laboratories, we may infer the national performance for *KRAS* testing from the MOKAECM study.

## Materials and Methods

### Study Design

This study was designed to evaluate *KRAS* genotyping in 40 French laboratories related to one of the 28 molecular genetics centers, using cell line and formalin-fixed paraffin-embedded (FFPE) tumor samples. ADNs were centrally prepared to control homogeneity and blindly sent to all participants for *KRAS* testing using routine practice technologies. Results were loaded and stored in a specific database and analyzed by a statistician (GC) from the HEGP hospital Clinical Research Unit.

### Cell Lines

ATCC Cells lines (H1573:p.G12A; H358:p.G12C; A427:p.G12D; LS123:p.G12S; SW620:p.G12V; Lovo:p.G13D; SW46:Wild Type) were specially purchased for the study and *KRAS* G12R, was obtained by retroviral infection of 292FT cells with a vector containing the *KRAS* c.34G>C substitution (JCP).

### Colorectal Cancer Tissues Samples

Twenty-four tumors were characterized and selected from patients undergoing surgical resection for colorectal cancer at the Ambroise Paré Hospital, (Boulogne-Billancourt, France). The Ethics committee of Ile de France II approved the study and patients were informed and written consent was obtained according to French law. The study was conducted in France. Diagnosis of colorectal adenocarcinoma was assessed by a pathologist (JFE) who selected the FFPE blocks for subsequent molecular analysis. DNA extraction was done at the Ambroise Paré Hospital using the Qiamp DNA Mini Kit (Qiagen). The tumors were characterized for *KRAS* by three different labs using three different technologies. These laboratories were selected based on their experience with KRAS testing and in house validation of the method used. Selected samples showed no discrepancy.

### Assessment of Cellularity

The evaluation of cellularity was performed at Hematoxylin, eosin and safran (HES) slides at both ends of the FFPE block used for DNA extraction. Slides were scanned on a Mirax Scan, (Zeiss, Göttingen, Germany). To validate the tumor cell content, the scanned images were reviewed by seven independent pathologists from different centers. When discrepancies were noticed, slides were reviewed and consensus was found.

### Methodology Used

Different in-house methods were developed: Sanger direct sequencing (n = 15 laboratories), allelic discrimination probe systems [8], [9] (n = 13), Snap Shot [10] (n = 7), pyrosequencing [11] (n−5), HRM followed by sequencing [12] (n = 5). One laboratory used the TheraScreen®: K-RAS Mutation Kit commercial kit. Five laboratories tested more than one method.

Thirty laboratories brought their entire protocol for *KRAS* mutation detection, there was no practice homogeneity except for laboratories using *KRAS* TaqMan® probes (see Information S1). Detailed procedures with primers positions are available on request.

### Statistical Approach and Data Analysis

Error rate was defined as the sum of false positives, false negatives, non-contributive tests and wrong mutation calling. To fit to the criteria used by the European EQA, all errors were at first considered equally significant although the implication for patients may differ. All samples were selected to have no amplification default and each participant submitted a result that we considered as being the final report sent to the oncologist. In a second step we considered clinically relevant errors as being false positive and negative results considering that in case of failure a new sample would be requested although this will result in a further delay for the patient.

Success rate was defined as the sum of true positives and true negatives.

We assessed both reproducibility (inter- and intra-laboratory) and diagnosis accuracy (sensitivity and specificity) of the different techniques for *KRAS* genotyping. For each mutant cell line, there were four different dilutions (5%, 20%, 50%, 100%) with three replicates per dilution. All the data were taken into account.

For the six techniques, used by at least two laboratories, inter-laboratory reproducibility was assessed using a generalization of the Cohen Kappa statistics for measurement of agreement among multiple rates [13]. In fact, each of the 96 samples was rated by *m* laboratories – with *m* ranging from 2 to 15 according to the technique- into one of the eight mutually exclusive categories. As there were failures (inability for the technique to give a mutation status), the calculation taking into account missing data. Confidence intervals for the true generalized Kappa coefficient were computed using the bootstrap resampling method, to take the intra-cluster correlation into account [14].

For assessing intra-laboratory reproducibility for a given *KRAS* genotyping technique, we computed a Kappa statistic for each laboratory, as described above, based on the triplicate aliquots. Then, we summarized the results by providing average Kappa coefficients, with the range of Kappa coefficients across laboratories.

Diagnosis accuracy for the detection of each specific mutation (categorical gold standard) was assessed for each technique and each laboratory. Materials used in the two rounds can be considered as gold standard materials (cell lines, validated tumor DNAs). It was possible to assess a sensitivity and specificity for each laboratory with cell line material and FFPE DNA samples. For each technique, sensitivity and specificity for the detection of mutation (binary gold standard) were computed by combining data from all laboratories. A ratio estimator for the variance of clustered binary data which takes intra-cluster correlations into account was used for calculating 95%CI [15]. All analyses were performed using the SAS software version 9.1.

### Economical Assessment

Five technologies were compared: “Direct sequencing”, “SNaPshot”, “Pyrosequencing”, “High Resolution Melting” (HRM), “TaqMan®”. Costs were estimated from the point of view of the laboratory by microcosting and time-motion studies. We estimated fixed and variable costs associated with each of the five technologies: labor, consumables (i.e. reagent and others consumables) and equipment and excluded overheads. Purchase price was used for supplies and equipment with a 5-year linear amortization and labor was valued using total payroll [16].

### Cost Sensitivity Analysis

A sensitivity analysis was led to get a range of cost by moving different parameters. The parameters were on prices (5% and ±10%) and laboratory number of acts (Information S1).

## Results

### Analysis of Cell Line Results

Ninety-six cell-line DNA samples were sent to 40 French laboratories, five laboratories used two different screening methods leading to 4320 reported results. Since 6 laboratories could not technically detect the p.G12R mutation (Information S1), the p.G12R samples were not taken into account and 3780 results were finally analyzed. Results were compared to the expected genotypes. The global error rate was 10.6% (399/3780), mainly due to false negative results (89%, 357/399). Among those, 307 tests corresponded to a percentage of tumor cells of 5% while 50 involved samples with higher tumor cell ratios. The 42 remaining errors were analytical failure (n = 25; 6%), false positive (n = 2; 0.5%) and wrong mutation (n = 15; 4%). Sequencing generated all false positive results and demonstrated a false positive rate of 0.4% (2/540). Wrong mutation callings were noted for sequencing (0.8%, 11/1260) and snapshot (0.7%, 4/588).

For techniques performed by more than 2 laboratories, sensitivity ranged from 76% to 96% and specificity from 95% to 100% (Table 1). Concerning 5% tumor samples, the lowest sensitivity was found for sequencing and HRM with an overall detection rate of approximately 40% as compared to 89.7% found for pyrosequencing. A technical failure rate of 1.6% was observed for Taqman probes due to a non-interpretation by 3 laboratories of the triplicates corresponding to p.G12V (SW480 100%) homozygous samples. Intra-laboratory and inter-laboratory reproducibility were in almost perfect agreements (>0.8 for all methods) and did not depend upon mutation type (Table 2).

**Table 1:** Diagnostic value comparison between methods cell line DNA analysis.

	Labs (n)	Samples (n)	Analytical failures (n)	Success rate in truenegative (%)	Success rate in true positive (%)				
					Dilutions				
					All	100%	50%	25%	5%
Direct sequencing	15	1260	4 (3‰)	98.9	76	99	99	87.0	38
Taqman	8	672	11 (1.6%)	99.0	92.3	95.8	100	99.3	76.4
Snapshot	7	588	4 (7‰)	98.8	89.7	95.2	100	93.7	73.8
Pyrosequencing	5	420	6 (14‰)	95	96.6	100	100	100	89.7
HRM and sequencing	5	420	0	100	78.0	100	98.9	88.9	40.0
MASA	1	84	0	100	92.5	100	100	100	72.2
Scorpion	1	84	0	100	100	100	100	100	100
HRM+Taqman	1	84	0	100	100	100	100	100	33.3
PNA° based methods*	1+1	168	0	100	100	100	100	100	100

°PNA Peptide nucleic acid; *PNA was used with taqman probes or with allele specific PCR.

**Table 2:** Intra and Inter-laboratory reproducibility on cell line DNA.

	Labs (n)	Samples (n)	Intra-laboratory Kappascore (CI 95%)	Inter-laboratory Kappascore (CI 95%)
Direct sequencing	15	1260	0.93 [0.79–1]	0.86 [0.84–0.87]
Taqman	8	672	0.97 [0.92–1]	0.93 [0.93–0.94]
Snapshot	7	588	0.97 [0.97–1]	0.88 [0.86–0.89]
Pyrosequencing	5	420	0.98 [0.94–1]	0.95 [0.94–0.96]
HRM and sequencing	5	420	0.97 [0.94–1]	0.86 [0.85–0.87]

### Analysis of Tumor Sample Results

Concerning FFPE tissues, 1128 data were generated and analyzed from 24 individual tumor samples (n = 47 methods, 40 different laboratories). The global error rate was 1.8% (20/1128) with 1 false positive, 7 false negatives, 9 analytical failures and 3 wrong mutation callings. Individual tumor samples correct calls ranged from 100% to 76.6%, indeed all laboratories correctly genotyped 16/24 samples and one sample (a *KRAS* p.G12A sample) generated mistakes by 11 laboratories. The 6 wild-type samples were correctly genotype except in one case were a false positive was reported by allelic discrimination qPCR based method with a PNA blocker. Thirty-two out of 47 result sets (laboratory/method) generated 0 error (68%), 13 made 1 (28%), 2 made 2 and one made 3 (Table 3). The three errors were 3 analytical failures using a pyrosequencing assay. This laboratory also used direct sequencing with one false negative result. If clinically relevant alterations (false positive and negative) and if best results are considered when more than one method was tested, 82.5% of the laboratories made no error and the success rate ranged from 100 to 91,6. The remaining errors were 6 false negative and one false positive in 7 laboratories. (Table S1).

**Table 3:** Global error rate for FFPE samples callings.

	Labs (n)	Samples (n)	Errors
Direct sequencing	13	312	12(3.85%)
Taqman	9	216	0
Snapshot	6	144	1 (0.69%)
Pyrosequencing	7	168	3 (1.79%)
HRM and sequencing	6	144	2 (1.39%)
Others	6	144	2 (1.39%)
Total	47	1128	20 (1.74%)

### Costs Per Item

Microcosting was assessed in site (n = 10 laboratories) by an independent team of health economists. Costs are given per item per test and total per test (Table 4). First labor costs ranged from €3.7 (TaqMan) to €11.4 (SnaPshot) per test due to different handling durations per sample from 7.2 (TaqMan) to 22.1 minutes (SNaPshot). Small differences between laboratories using identical technology were observed owing to slight variation in protocols and to different equipments that influence efficiency and labor costs. Moreover the number of samples run per batch also induces labor cost variation. Second, equipment costs per test ranged from €1.0 to €9.7 depending on laboratories and technologies. Direct sequencing was the most expensive technology with more than €7 per test. Pyrosequencing, HRM and TaqMan were the least expensive technologies with less than €2 per test. Sequencer, Pyrosequencer and qPCR thermocycler generated 84% of equipment costs. Machine costs per test varied according to duration of runs, purchase prices and maximum number of samples per batch.

**Table 4:** Costs per item and total costs per test per technique and laboratory.

Techniques	Sequencing		HRM		SnaPshot		Pyro-sequencing		TaqMan	
**Laboratory #**	1	2	1	2	1	2	1	2	1	2
**Labor (€)**	9.9	8.0	3.9	6.4	11.4	8.7	7.7	8.5	5.5	3.7
**Equipment (€)**	9.7	7.7	1.0	1.5	4.3	5.2	1.7	1.4	1.4	1.3
**Consumables (€)**	9.8	10.1	9.6	6.4	19.0	6.4	12.1	11.2	6.1	5.5
**Total costs (€)**	29.4	25.8	15.0	13.9	34.8	20.3	21.6	21.1	13.1	10.6

Third, consumables prizes per test ranged from €5.6 to €19.0. Number of replicates, kind of reagents used, technical processes and price negotiations explained most of the differences observed from laboratories using the same technology. These differences were particularly important for SnaPshot technique. One SnaPshot laboratory replicated experiments and used more expensive reagents, leading to threefold higher consumable costs compared to the second laboratory studied.

### Total Costs

Total costs per test ranged from €10.6 to €34.8 (Table 4). Moreover total cost for HRM needs to take into consideration sequencing costs. About two thirds of samples were detected as wild type KRAS genes by HRM and did not require sequencing. We observed a rise of post “HRM” sequencing costs from €7 to €13 compared to direct sequencing costs despite similar technologies. Therefore the global total cost per HRM test ranged from €27.0 to €28.0.

### Cost Sensitivity Analysis

**Figure 1:**
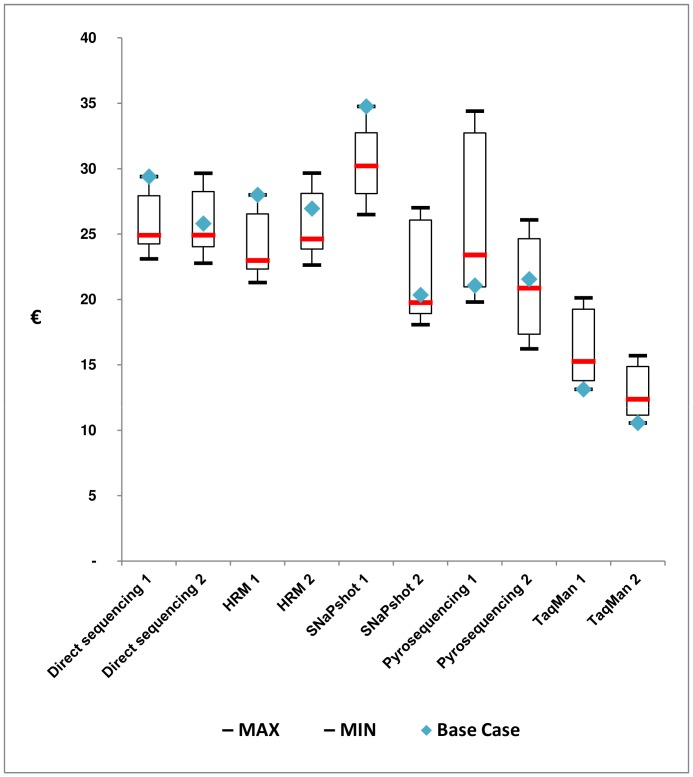
Sensitivity analysis; Minimum, maximum and base case costs per test per technique and laboratory. Bleu diamonds rare base case costs.

Sensitivity analysis confirmed that TaqMan was less expensive than other technologies (Figure 1). The estimated costs for TaqMan were optimal within the base case. Within the worse conditions (five samples per batch and 10% prices markup) TaqMan prices per test ranged from €15.7 to €20.1. Pyrosequencing costs per test were lower than 20.1€ if at least 15 samples per batch were run.

## Discussion

The use of anti-EGFR monoclonal antibodies is restricted to the 60 to 70% KRAS wild- type metastatic colorectal tumors, making appropriate identification of KRAS mutations a key point for clinical practice [17], [18]. Moreover limiting the use of EGFR inhibitors to patients with wild-type KRAS may be a potential solution for cost savings [19]. To ensure testing accessibility, the INCa has granted 28 regional molecular genetics centers up to 2.5 M€ [20]. The aim of the countrywide quality control network named MOKAECM, supported by the INCa, was to evaluate the different in-house developed methods by molecular laboratories for KRAS testing in routine conditions. Here, similar cell lines (4296) and FFPE tumor samples ADNs (1152) were sent to the different participants and 5448 KRAS genotyping results were submitted and analyzed. Concerning the cell lines test, the error rate was 10,6%, the detection cutoff ranged from 5 to 20% and 86% of false negative were related to the 5% dilution. 1152 FFPE sample results were analyzed in this study, 68% of test sets (method/laboratories) correctly identified the KRAS mutational status in all FFPE samples. This result is comparable with the score reported in the European KRAS EAQ scheme (70%) [21]. Recently, for KRAS mutation analysis in colorectal cancer, arbitrary thresholds for correct *KRAS* mutation identification was set at 97% [22]. Considering best results for laboratories testing more than one method, the average success rate for FFPE samples was 98.5 and ranged from 100 to 91.6% (Information S1). These scores are in accordance with the results of a study ran in 10 laboratories in Netherlands [23]. Moreover, failure to attain an overall testing event score of at least 80% was defined unsatisfactory when testing a larger number of cases in the ‘Clinical Laboratory Improvement Act’ of 1988. Taking into account results from the two testing sets 96% of French laboratories had a satisfactory score over 80% that clearly shows the quality of the KRAS testing in France despite the large panel of methods. Moreover among in the 1152 tumors tested, only 20 errors were reported. This level of error is satisfying.

From a national perspective, on 1152 tumor tests in this study, 20 errors (0.017 CI95% [0.01–.027]) were reported with 11 of them related to a single sample. Therefore the corrected error rate was 0.7% CI95% [0.03%–0.13%] per test and per tumor. An extrapolation suggests that in France, out of the 18,000 tumors analyzed each year, 54 to 234 tumors could be misgenotyped.

Genotyping errors can result from different issues as the type of fixative, the preservation procedure, the evaluation of the tumor-cell content and finally the performances of the method used for testing. Here, DNA extraction was centralized and tumor cell content was validated by 7 independent pathologists therefore only *KRAS* genotyping methods were compared. All selected samples had a first validation of their KRAS status carried out by three reference laboratories using three different methods. Many different techniques, including commercially available kits, have been developed and tested but the absence of a recognized reference method makes the evaluation of new technologies a difficult task.

Cell line testing may not reflect routine practices but was used as a validation test in optimal conditions to compare the sensitivity and specificity between the different technologies. For samples with tumor cell line content over 20%, that can be considered clinically relevant, analytical results showed almost perfect agreements. For samples under 20%, results were more heterogeneous, especially for direct sequencing. In our experience, HRM prescreening did not rescue low tumor content samples. The lower sensitivity of sequencing methods as compared to others was not a surprise [24], [25], [26], but our results also point out that performance might depend on method optimization and level of expertise. Indeed 7 laboratories using sequencing or HRM-sequencing had a low error score (<10%) in the cell line series including 5% cell line samples and no error in the tumor series. Sensitivity seems related to a couple - methodology/laboratory-experience - rather than strictly to a method, therefore validation and detection cutoff must be assessed in each laboratory. When low sensitivity methods are used for genotyping, macrodissection cutoff must be adequately chosen and clear preanalytic recommendations must be given to pathologists before DNA extraction. Regardless of the detection method, mutation type could impact on sensitivity. The rate of errors was around 3% with the p.G12V to more than 20% with p.G12S and p.G12A. Allelic quantification of the 7 cell line DNAs using pyrosequencing did not give any relevant explanation to the reduced sensitivity observed for some mutations, and thermodynamic consequences in the DNA melting behavior might, in part, explain this observation. In the FFPE series the error rate was 20/1128 (1.7%) versus 399/3780 (10.6%) in cell lines, for which errors were mainly due to 5% tumor cell false negatives. The error rate was 2.6% for cell lines in similar tumor content conditions (false negative due to 5% samples excluded) suggesting that tissue fixation was not an obstacle to *KRAS* testing. However, fixation could slightly impact on failure levels: 0.8% (9/1128) on FFPE samples versus 0.6% on cell line DNAs (25/3780). The use of methods based on the amplification of small amplicons could be a possibility to decrease the level of non-contributive results [27]. In this study, methods based on allelic discrimination based on small fragments amplification and real time PCR demonstrated no failure against up to 4% for direct sequencing methods. Finally, in the FFPE series, 14% of the errors were found in a single sample for which tumor cell content was 50% after HES examination but the quantification of mutated alleles by pyrosequencing suggested that mutant cells could only represent 20% of all. This might in part explain the discrepancies observed for this sample but also suggests that genetic variations are not equally detected. One FFPE false positive sample was identified by allele specific amplification with a PNA blocker. It was not validated by another laboratory using similar technology and was therefore considered a false positive. Moreover the clinical value of mutated sub-clone remains to be demonstrated [28], [29], [30], [31], [32].

### Cost Estimates Limitations

This methodology of assessment of the cost was single handed since the same independent team assessed all the technology directly in the laboratories. Five technologies were studied by microcosting in ten laboratories representing one third of all French “platforms”, which carried out 25% of all *KRAS* mutations tests for metastatic colorectal patients in France in 2009. Although, the level of evidence could be suboptimal, as it was based on 2 observations per technology, allelic discrimination using TaqMan probes was about two or three times less expensive than any other technology studied. Cost variation within one technology or between technologies could be due to different procedures. Indeed methods were not strictly identical, even in laboratories using similar technology, and various degrees of optimization were reported. An example was the management of testing procedure – the number of positive and negative controls, the number of replicates and the number of added steps to the procedure such as gel migration of PCR products. These extra costs were valued. Concerning cost equipment a saturation hypothesis was set at the rate of eight hours per working day during five years. This could not be the true situation for some laboratories and the estimated costs underestimated true costs for laboratories. Moreover, according to the saturation hypothesis of equipment, it is assumed that the life expectancy of machines was similar for any kind of machine, no information was available on the real life expectancy of machines. Altogether microcosting data suggested that “in-house” technologies costs were much lower than commercial kits, excluding equipment and labor costs.

### Conclusion

The French population was 65,027,000 of inhabitants in 2011. Twenty-eight regional molecular genetics centers covering all the territories and coordinating 46 laboratories are now involved in the analysis in 16,000 *KRAS* testing for metastatic colorectal cancer each year. The whole network is nationally managed by the INCa. This quality control program was the first countrywide experience with 120 similar samples being analyzed by 40 different laboratories. Our results demonstrate that, when clinically relevant results are considered 82,5% of laboratories correctly identified the *KRAS* mutational status in all FFPE samples. This work also suggests that, while all methods are suitable for *KRAS* testing with an average cost of €35 per test excluding the preanalytical steps, differences exist in terms of sensibility and robustness. The choice of the method is likely to depend on the equipment and technical expertise available locally. This quality program provided a baseline picture of *KRAS* testing in France. It showed that it is possible at a reduced cost to set a nationwide program, it identified errors in testing procedures for some laboratories underlining the importance of optimization, in-house validation and quality control processes using a large panel of mutations.

## Supporting Information

Information S1 **Details of material and methods.**
(DOC)

Table S1 **Shows the detailed genotypes information for FFPE samples by laboratory (N = 40).** The best *KRAS* results were kept for laboratory using more than one technology.
(XLSX)
