# Supplementary material for: Performance and Cost Efficiency of KRAS Mutation Testing for Metastatic Colorectal Cancer in Routine Diagnosis: The MOKAECM Study, a Nationwide Experience
Source: PLoS One. 2013 Jul 25;8(7):e68945. doi: 10.1371/journal.pone.0068945 (PMC3723748; doi:10.1371/journal.pone.0068945)
Supplement: Information S1 — Details of material and methods. (DOC) [file pone.0068945.s001.doc]

**Supporting information**

Detail information concerning material and methods

Cell Line DNA extraction modalities

Cell line DNAs were extracted using the QIAamp DNA Mini Kit (Qiagen, Courtaboeuf, France) following the manufacturer’s instruction, quantified by spectrophotometry (NanoDrop ND-100 instrument, Thermo Fisher Scientific, Walthmam, MA), normalized to 30ng/uL and were kept at -20°C up to analysis. The seven cell-line DNAs were diluted in SW46 DNA (mutant/SW46: 1; 0,5; 0,25, 0,05). Each laboratory received a set of 96 randomly numbered vials consisting of the dilutions in triplicates for each cell line DNA and 12 aliquots of SW46 DNA. A total of 3600 samples were sent to 40 laboratories. Extraction, dilution and distribution were centralized in one laboratory (UMR775, INSERM, Pr P. Laurent-Puig).

The p.G12R cell line obtained by direct mutagenesis has a mismatch located on codon 28 (c.84 T>C). It was subsequently shown that laboratories that did not correctly genotype the p.G12R had chosen their upper primer on the polymorphic locus. p.G12R results were therefore not considered to compare laboratories.

Colorectal cancer tissues samples

Tumors were selected with a minimum of 30% tumor cells in each sample as determined by collective assessment. DNA was extracted from formalin-fixed paraffin-embedded tissue (FFPE) using the QIAamp DNA Mini Kit (Qiagen, Courtaboeuf, France) according the manufacturer’s instructions.

A reference genotype was defined for the FFPE samples by a pre-screen done by five laboratories using five different methods (HRM-sequencing, direct sequencing, pyrosequencing, snap shot and allelic discrimination using TaqMan® probes).

Nineteen tumors found to have the same KRAS status were then used to prepare 24 tumors DNA samples randomly registered (vial 1 to 24) and normalized to 30 ng/µL. Fourteen of the 19 samples were macro-dissected to contain more than 50% tumor cells. Five of the 19 samples were used to produce two preparations, one macrodissected (>50% tumor cells) and one non-macrodissected (30-50% tumor cells).

Technologies

*Didesoxy sequencing*

The automated Sanger sequencing was performed in 15 laboratories. The purification process was the ExoSAP-IT protocol (GE Healthcare) in most centers. Different Big Dye Terminator versions were used from V1.1 to V3.1 (Applied Sciences). Sequencers were ABI3100, 3130XL, 3730 (Applied Sciences) and one laboratory used a Beckman 8 capillary CEQ8000 (Beckman).

*Probe based technologies*

TaqMan® probes were performed by eight laboratories. *KRAS* mutation status was assessed by means of the same set of primers and methodology in the laboratories but different thermocyclers were used ABI7900 (Applied Sciences, Courtaboeuf, France), LightCycler480 (Roche Applied Sciences) and iCycler iQ (BioRad). Probe and primers sequences are available in Didelot et al. [Exp Mol Pathol.](http://www.ncbi.nlm.nih.gov.gate2.inist.fr/pubmed?term=didelot blons) 2012;92:275-80.

Other allele discrimination methods were developed. One MASA in house method, one real time PCR with PNA blocker, 1 TaqMan probes with PNA and 1 center used the Scorpion commercial kit (DxS).

*Pyrosequencing*

Pyrosequencing was applied in five laboratories. One laboratory used the *KRAS* detection kit from Biotage. The materials were PyroMarkMD or PyroMarkID (QIagen).

*HRM-sequencing*

The HRM analysis and sequencing was proposed in five laboratories. Tumor DNA was mainly analyzed using LightCycler 480 real-time PCR instrument (Roche Applied Science, Meylan, France). The dye used was either LCgreen (Idaho Technologies, Salt-Lake City, US) or Resolight (Roche Applied Science) in the HRM Master mix (Roche Applied Science).

*Snap Shot*

Snap Shot was proposed in seven laboratories. Several types of sequencers were also used as ABI3100, or 3130XL (Applied Sciences).

*Economical assessment*

***Micro-costing method***

Five technologies were compared: “Direct sequencing”, “SNaPshot”, “Pyrosequencing”, “High Resolution Melting” (HRM), “TaqMan®”. Costs were estimated from the point of view of the laboratory. We estimated costs associated with each of the five technologies. Overhead and pre-analytical steps (e.g. pathological examination, DNA extraction) costs were not taken into consideration

We identified three costs categories: labor, consumables (i.e. reagent and others consumables) and equipment. We used a micro-costing method based on direct observation. Ten laboratories (two for each technology) were visited in order to observe procedures, to create inventories of materials and consumables used and to collect price information and labor and machine times.

Costs were estimated in the context of the laboratories visited. Since the tests are carried out in batches, a cost per batch was estimated that we divided by the number of samples in the batch in order to calculate a cost per test.

Labor costs were valued using handling duration and the median gross wage of biomolecular laboratory technicians of the visited laboratories. Consumable costs were valued using real consumptions and real purchase prices. Equipment costs were valued using utilization duration and real purchase prices. The saturation hypothesis of equipment was set at the rate of eight hours per working day during five years. The saturation hypothesis of machine runs was set. Median prices for common consumables and equipment (e.g. pipettes, tubes) and for costly machines (e.g. sequencer, pyrosequencer) were employed. When consumables or equipments real purchase prices were not available we used catalog prices with a 20% discount.

*Cost sensitivity analysis*

A sensitivity analysis has been conducted regarding prices data and number of samples. Real purchase prices, or median purchase prices for few references, have been used. Since purchase prices arise from negotiations and unequal bargaining power, equipment and consumables costs estimates were possibly biased with relevant price variations between laboratories. The two laboratories carrying out the TaqMan® technologies were linked-up with the Assistance publique Hopitaux de Paris INCa “platform” which performed about 20% of *KRAS* mutations tests in 2009 which can result in a greater bargaining power. A +5% and +10% price variations have been tested for TaqMan® laboratories. A -5% and -10% price variations have been tested for the other eight laboratories.

The number of samples varied from observation to observation. However, economies of scale affect the cost per test estimates. In terms of labor time, some steps of the process have a fixed duration whatever the number of samples was. The cost per test of these steps strictly depends on the number of patients. In terms of consumables, the number of control samples was fixed whatever the number of patient samples was. The cost per test of these control samples strictly depends on the number of patients. Therefore, the comparison of human resources and consumables costs was possibly biased. In terms of equipment, the bias in machine cost estimates was marginal as the saturation of run hypothesis was used. Numbers of samples have been tested by simulations. Two scenarios beside the base case (cost estimate with observed conditions) with five, ten or fifteen samples were simulated. The variation rates estimates of human resources consumptions were based on a process analyzes. The steps with a fixed duration and steps with a variable duration were distinguished. The variation rates estimates of consumables were based on the variation rates of the number of well per patients (control samples well and patients samples well).
